# Parent psychological wellbeing in a single-family room versus an open bay neonatal intensive care unit

**DOI:** 10.1371/journal.pone.0224488

**Published:** 2019-11-05

**Authors:** Bente Silnes Tandberg, Renée Flacking, Trond Markestad, Hege Grundt, Atle Moen

**Affiliations:** 1 Department of Paediatric and Adolescent Medicine, Drammen Hospital, Vestre Viken Hospital Trust, Drammen, Norway; 2 Lovisenberg Diaconal University College, Oslo, Norway; 3 Department of Clinical Science, Faculty of Medicine and Dentistry, University of Bergen, Bergen, Norway; 4 School of Education, Health and Social Studies, Dalarna University, Falun, Sweden; 5 Department of Paediatrics, Haukeland University Hospital, Bergen, Norway; 6 Department of Neonatology, Oslo University Hospital, Oslo, Norway; Hopital Robert Debre, FRANCE

## Abstract

**Background:**

Studies of parents’ psychological well-being in single-family rooms in neonatal intensive care units have shown conflicting results.

**Aims:**

To compare emotional distress in the form of depression, anxiety, stress and attachment scores among parents of very preterm infants cared for in a single-family rooms unit vs an open bay unit.

**Study design:**

Prospective survey design.

**Subject:**

Parents (132) of 77 infants born at 28 0/7–32 0/7 weeks of gestation in the two units.

**Outcome measures:**

Duration of parental presence was recorded. Scores for depression (The Edinburgh Postnatal Depression Scale), anxiety (The State–Trait–Anxiety Inventory, Short Form Y), stress (The Parent Stressor Scale: neonatal intensive care unit questionnaire and The Parenting Stress Index—short form) and attachment (Maternal Postnatal Attachment Scale) measured 14 days after delivery, at discharge, expected term date and four months post-term.

**Results:**

Parents were present 21 hours/day in the single-family room unit vs 7 hours/day in the Open bay unit. Ninety-three percent of the fathers in the single-family rooms unit were present more than 12 hours per day during the first week. Mothers in the single-family rooms had a significantly lower depression score -1.9 (95% CI: -3.6, -0.1) points from birth to four months corrected age compared to mothers in the Open bay unit, and 14% vs 52% scored above a cut-off point considered being at high risk for depression (p<0.005). Both mothers and fathers in the single-family rooms reported significantly lower stress levels during hospitalization. There were no differences between the groups for anxiety, stress or attachment scores after discharge.

**Conclusion:**

The lower depression scores by the mothers and lower parental stress scores during hospitalization for both parents supports that single-family rooms care contribute to parents’ psychological wellbeing.

## Introduction

Parents of preterm infants often face immediate and prolonged separation from their babies during hospitalization. The post-partum emotional response of both the mother and the infant is rooted in instincts programmed by evolution to secure survival and safety of the mammalian off-spring, and separation may induce distress and fear in both[1, 2]. Compared to other mammalian species, the brain of the human newborn is larger and more adaptable, but also particularly immature and dependent on caregiving behaviours and a nurturing environment [3]. There is increasing evidence that early experience and stimulation may influence long-term outcomes and the mechanism may at least partly be related to the rapid development of the brain during infancy and most pronounced in infants born preterm [4]. Stressors during the neonatal intensive care unit (NICU) hospitalisation may affect regulation of the hypothalamic- pituitary-adrenal axis, which is our central stress response regulating system, as well as general brain development [5, 6]. The bonding and interaction between infants and their mothers are also important for healthy developmental trajectories [3]. Over the last two decades the principles of family-centred care have gradually been implemented in the care of premature and sick newborn infants [7, 8] and in 2018, the European Foundation for the Care of Newborn Infants (EFCNI) launched the European standards of care for newborn health, defining family-centred care and a physical environment that allows extensive parental presence and participation as the European standard of care for hospitalized newborn infants [9, 10]. Parental presence also brings care in accordance with the UN Convention on the Rights of the Child, Article 7, acknowledging the infant’s right to be cared for by his or her parents [11]. However, there are large variation between units in parental presence and involvement, also in units claiming to work in accordance with family-centred principles [12, 13].

The number of NICU’s with a single patient or single-family room design (SFR) is growing. The medical and psychological benefits of including parents in care have been well documented [14], and one study in particular has provided evidence for both short- and long-term medical benefits of SFR care [15–17]. Parents’ participation in care may also be beneficial for parents’ own mental health [14, 16]. However, Pineda et al. [12] and Domanico et al. (13) showed an increase in parental stress and isolation when infants were treated in single-patient rooms. Even though parental presence increased with a SFR design, the time of parental presence in these studies was low, in particular the time providing active care, holding and skin-to-skin contact (SSC) [18, 19]. Pineda et al. have even indicated adverse findings on MRI and neurodevelopmental outcome at two years after care in single-patient rooms [20]. However, in this unit single room care was carried out with very limited parental presence and family participation compared to what is commonly seen in a Scandinavian NICUs [21]. Although most NICU professionals may acknowledge that parent participation is warranted, there is no consensus on how much presence and active participation in care parents can and wish to provide. It is well documented that parents of preterm infants may experience mixed emotions, causing symptoms of stress, anxiety and/or depression [22, 23]. In this study, we have used parents’ self-reports of depression and anxiety, stress and negative influence on attachment as indicators of emotional distress.

Differences in parental outcomes may be influenced by external policy factors such as rights to parental leave and access to health insurance, and by socio-economic differences that are not directly observable by parents or the NICU staff. In addition, differences in infant morbidity between studies may contribute. No studies have explored the effects on emotional distress when both parents live with their infant all or most of the day from birth to discharge, and we therefore designed a controlled study of parents’ emotional distress in two different units providing care in accordance with the principles of family-centred care. One unit had a SFR design; the other was an old unit with an open bay (OB) design.

We hypothesized that parents participating actively in care through continuous presence in a SFR unit did not experience more emotional distress than parents in an OB unit who spent less time with their infant.

## Materials and methods

We have previously reported effects of SFR design on parental presence, infant growth trajectories, morbidity, medical procedures and nutrition [24]. In the present study, we report parents’ emotional reactions to continuous presence, using questionnaires to screen for the risk of depression, anxiety, stress and attachment, and we provide a more in-depth description of parental presence. Both participating units were located in maternity hospitals and provided care until discharge.

### The units

In 2012, the NICU at Vestre Viken Hospital Trust, Drammen, Norway, was established as a SFR, allowing parents to stay with their infant day and night from birth to discharge and to participate as primary caregivers. The unit provided care from birth for infants with gestational age (GA) ≥ 28.0 weeks and admits approximately 450 infants in 17 beds annually. Each room has two different areas; one infant-area with a place for the incubator or cot, sink, nursing table, and equipment (CPAP, pumps, ventilators), in addition to a parent-area with two high-quality hospital beds (105 cm wide electrically adjustable). Separate bathrooms are included in all SFRs. At day time, there is no physical separation between the parent and infant area and equipment are mounted on flexible arms allowing easy and secure transfer of the infant from the incubator to the parents’ bed without disconnecting medical equipment. During night time, parents can close flexible folding doors to the sleeping area, while nurses still have direct access to the infant without interrupting parents (pictures of the SFR unit are presented as supplement, S1a–S1c Picture). All meals were provided without cost to both parents. At the time of the study, parents had access to a psychologist working part time at the unit and to weekly parent meetings with other parents. The unit was staffed with five consultants having 50% of their clinical service in the unit and 62 registered nurses of whom 24% were specialists in intensive care, paediatrics, or neonatal nursing. Parents were present and participated actively during daily rounds.

The OB unit was located at Haukeland University Hospital in Bergen, Norway, and provided care from birth for infants with GA ≥ 23.0 weeks. The OB unit was built in 1979 and underwent no subsequent major changes. It had 21 beds and admitted approximately 500 infants per year. Except for one single-bed room used for high-intensive or end-of-life care, the unit had two rooms; one for intensive- and intermediate care infants and one for care in cots before discharge to home. The rooms were crowded, but one reclining armchair could be placed between incubators or cots, and screens could be placed around the family to provide some privacy. The parents had unlimited access at all hours, but they could not stay overnight in the unit. Mothers were offered accommodation in another building at the hospital area after discharge from the maternity ward. Free meals were provided only for mothers. A psychologist was available upon special request. The number of neonatologists in full time position was 3.5 and 64% of nurses were specialist nurses. Parents were not routinely involved in medical rounds.

Although the facilities available for parents to room in were different, both units had an explicit policy of allowing parents unlimited access and to stay with their infant for as long as they wanted. SSC was strongly encouraged in both units. Both units encouraged and guided mothers to provide breastfeeding from day one.

Norway has extensive social benefits related to pregnancy and birth. Health care insurance is publicly funded, hospital care is free of costs and both parents are allowed full job-leave with compensation for salary-loss during hospitalization with their infant. Parents also have 48 weeks of fully paid parental leave shared between them after discharge from the NICU.

### Participants

**Fig 1:**
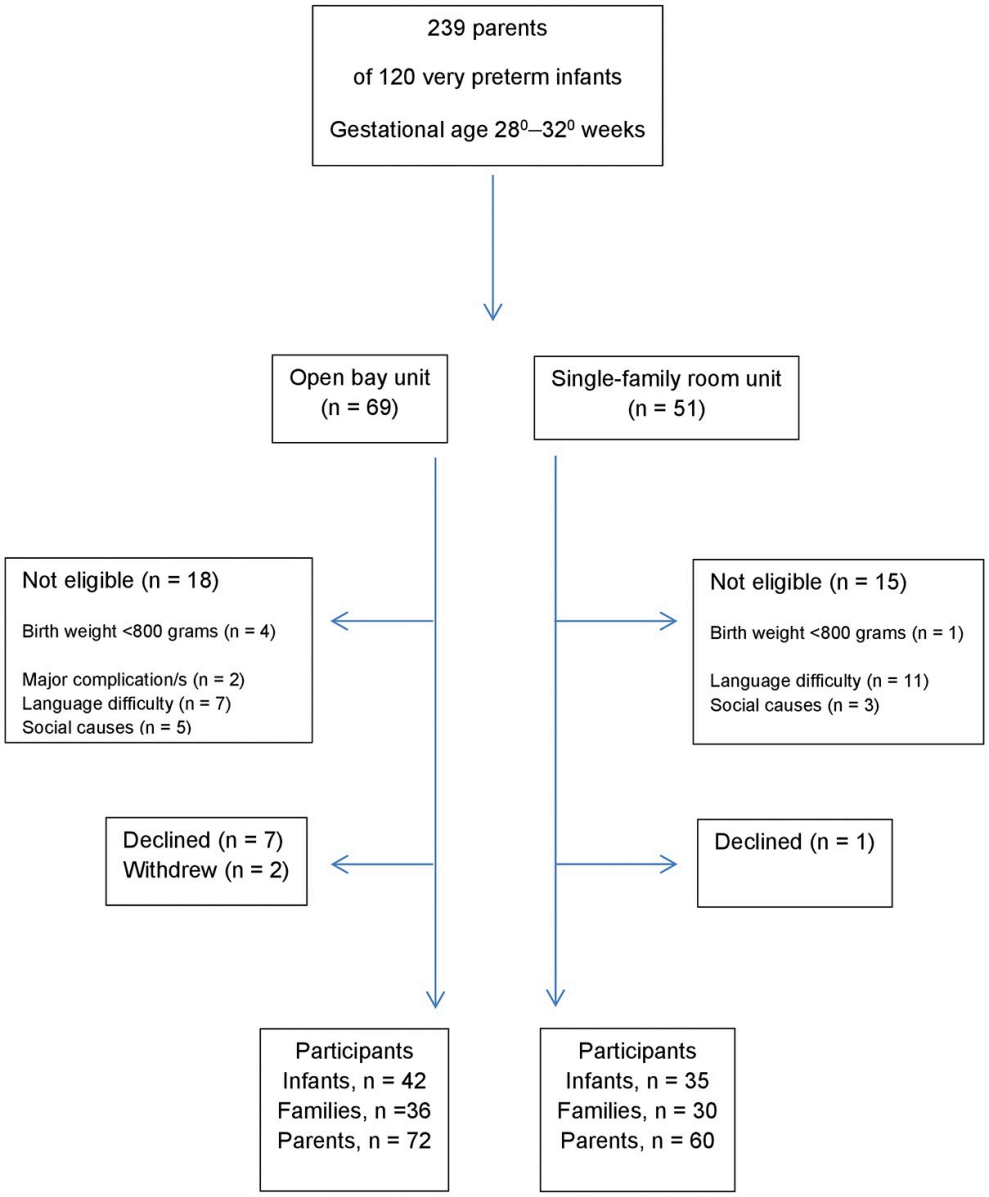
Flow diagram of participant recruitment.

Parents of infants born at 28 0/7–32 0/7 weeks of gestation with the mothers’ address in the hospitals’ respective catchment areas were eligible for inclusion. Infants with congenital malformations or major complications (intraventricular haemorrhage grade III/ IV or surgically treated necrotizing enterocolitis) and infants with birth weight less than 800 grams were excluded. We also excluded infants if one or both parents suffered from a major mental illness or did not understand Norwegian language, infants of mothers who had taken illicit drugs or were on methadone during pregnancy and infants in the custody of the Child Protection Services from birth. Both parents received oral and written information about the study, and they were included if both gave written consent by the end of the second day post-partum. In the SFR unit, 60 parents of 35 neonates were included and in the OB unit 72 parents of 42 neonates were included consecutively. Inclusion to the study started on May 1, 2014 and ended on July 31, 2016 as the OB unit was moved to another building with better facilities (Fig 1).

The study was approved by the Norwegian Regional Committee for Medical Research Ethics and registered at ClinicalTrials.Gov (NCT 02452580).

### Data collection

For each infant, both parents prospectively recorded time present in the unit with their infant and the duration of SSC on the mother’s or father’s bare chest. From birth to postmenstrual age of 34 weeks, both periods were registered each day in a closeness diary lying next to the infant. Twins had separate diaries. Continuous presence was defined as presence for more than 12 hours a day for each parent.

Parents were asked to complete a set of questionnaires at 14 days post-partum, at discharge, at term date and at four months after term date. If one parent did not participate in a follow-up consultation after discharge, the questionnaires were brought home with the participating parent along with a stamped envelope and returned by post to the project manager. For twins parents answered one set of questionnaire.

1) *The Edinburgh Postnatal Depression Scale (EPDS)* [25] aims to identify depressive symptoms in pregnant women or women who have recently given birth. The EPDS is validated for use in a Norwegian population [26, 27]. The range of score is 0–30 and the score increases with a increasing symptoms. We applied a cut off score ≥ 13 giving a sensitivity of 77% and specificity of 94% in detecting symptoms of depression [28].

2) *The State–Trait–Anxiety Inventory*, *Short Form Y (STAI SF)* measures symptoms of anxiety in adults [29]. The short version contains six statements, three items with anxiety present and three with anxiety absent, which the respondents rate on a scale from 1 to 4 [30]. The range of the total STAI score is 20–80 and it increases with increasing symptoms. Scores below 36 is considered normal [31]. STAI SF has demonstrated reliability and validity in study samples of parents with sick infants [32].

*3) The Parental Stressor Scale*: *NICU (PSS*: *NICU)* [33, 34] measures stress experienced by parents during hospitalization related to alterations in their parental role, the appearance and behaviour of their child, and sights and sounds of the unit. Parents are asked to rate items on a five-point scale ranging from "not at all stressful" to "extremely stressful". “Sights and sounds of the environment” and “Infant’s appearance” are scored as one sub-scale, with scores ranging from 20 to 100. “Parental role alteration” has a range of scores from 7 to 35. The tool has been shown to predict depressive symptoms [34] and a moderate correlation with state anxiety [35] and has also been validated for a European population [36].

4) *Parenting Stress Index (PSI- SF)*. The short form (36 questions) of PSI is a widely used clinical and research self-report questionnaire to identify stress due to parental factors or deviant development of the child [37, 38]. The questionnaire includes a parent domain (i.e. social isolation, attachment to the child, health, role restriction, depression and partner) and a child domain (i.e. distractibility/hyperactivity, adaptability, how demanding the child is perceived to be, mood and acceptance). The total score ranges from 18 to 90 and higher scores indicate higher levels of parent-related stress. A total score between 52 and 90 is considered to represent a high-risk level, whereas scores from 18 to 44 are considered low-risk/normal [39].

5) *The Maternal Postnatal Attachment Scale* (MPAS) evaluates the mother’s subjective feeling of attachment (“the emotional tie”) to the infant (40). In this study, fathers also were asked to complete the MPAS. The instrument consists of 19 statements referring to three different factors: *patience and tolerance*, *pleasure in interaction* and *affection and pride*. The respondents indicate to what extent (always, very often, often, sometimes) the statements match their perception. The possible range of scores is 19 to 95, higher scores indicating more attachment. At term date, the mean normal score was 83 (range 56–95) and at four months post-term it was 85 (range 59–95) [40].

The STAI and MPAS tools were translated into Norwegian with forward and backward translation. For PSS: NICU, a former Norwegian translation was used.

Questionnaires were not returned by: 2% and 13% of the mothers and fathers, respectively, at day 14; by 3% and 16% at discharge; by 18% and 27% at term; and by 20% and 17%, respectively, at four-month corrected age. There were no significant differences between the two groups in the number of unreturned questionnaires.

### Statistics

The SFR and OB units were compared by independent sample t-tests, Mann-Whitney tests or Pearson’s chi-square tests, according to distribution of the data. Two baseline characteristics were unequally distributed in the groups (mode of delivery and education). Therefore, in addition to the main explanatory variable (the SFR or the OB unit), mean differences in outcome measures (parents’ answers to the questionnaire) were analysed with a linear mixed model. This model included repeated measurements and thereby the effects of time, and took into account the correlation structure and dependency between the repeated measurements. The model treated each of the measurements (scores from the different questionnaires from birth to four months after expected term date) as level one and the individual parent as level two. This is a two-level model with fixed effects for unit, time, mode of delivery (vaginal or caesarean section) and parental education (elementary, high school and college/university). In the mixed model, we used the autoregressive covariance structure (AR1) because the correlations between adjacent time points were higher than the correlations between measurements at time points further apart. Model assumptions (collinearity, residuals and outliers) were thoroughly checked. Results were given as an estimate of the mean difference between the OB and SFR units, adjusted for confounders with corresponding 95% confidence intervals. For the questionnaires, EPDS, STAI and MPAS missing values of the inventory were replaced by the mean value from remaining items when no more than one was missing from the sub scale.

One item on each of the two different sub-scales in the PSS: NICU (“Sights and sounds of the environment and Infant appearance”; and “Parental role alteration”) were systematically missing because of a technical failure when distributing this questionnaire to parents in both units. The two items (“My baby’s unusual or abnormal breathing patterns” and “Not being able to hold my baby when I want”) were replaced by the remaining items on each of the sub-scales, after agreement with the author, Dr M. Miles (e-mail correspondence dated 05.09.2018). For the PSI, which contains several domains, answers were replaced with the mean value from the other score within each domain if no more than two item answers were missing from the parent domain and no more than one item from the child domain. The statistical significance was set at a p-value of <0.05.

Mean differences between the SFR and OB units in duration of parental presence and SSC until postmenstrual age of 34 weeks were determined in linear regression analyses. The main exposure was the unit (SFR or OB), and the outcomes were adjusted for postmenstrual age at birth, mode of delivery (vaginal or caesarean section) and parents’ education (elementary/high school or college/university). Analyses of parental presence were performed separately for mothers and fathers, with an additional analysis of the cumulative parental presence and SSC for each infant. All analyses were done in SPSS Statistics version 25 (IBM, Inc., Armonk, NY, USA).

## Results

The SFR parents had a lower level of education, a higher proportion of the infants were delivered by caesarean section and their mean GA was slightly higher (Table 1).

**Table 1:** Characteristics of the families and infants cared for in the single-family room (SFR) and open-bay (OB) units presented as means (SDs) or number (%) within each unit.

Variable	SFR unit	OB unit	*p-*value^1^
	(n = 35)	(n = 42)	
Mother’s age (y)	31 (7)	32 (6)	0.38
Father’s age (y)	36 (10)	34 (7)	0.45
Single mother, n (%)	0 (0)	1 (2)	0.66
**Norwegian first language**, n (%)			
Mother	28 (80)	39 (93)	0.21
Father	30 (86)	39 (93)	0.30
**Education level**^2^**,** n (%)			
Mother			
Elementary	4 (13)	0 (0)	0.015
High school	10 (33)	10 (30)	
College/university	15 (50)	23 (70)	
Father			
Elementary	3 (10)	0 (0)	0.012
High school	15 (50)	12 (38)	
College/university	12 (40)	20 (63)	
**Infant**			
Caesarean section, n (%)	25 (71)	20 (48)	0.04
Primipara, n (%)	8 (23)	11 (34)	0.64
Gestational age (GA) (min, max)	30.5 (28.2, 32.0)	30.1 (28.1, 31.6)	0.03
PMA^3^ discharge, days	252 (9)	255 (14)	0.34
**Parental presence**			
Mother			
First week, hrs	111 (38)	33 (13)	<0.001
Overall average presence^4^, hrs	21 (5)	7 (3)	<0.001
Continuous presence^5^, n (%)	26 (87)	0 (0)	<0.001
Father			
First week, hrs	115 (39)	31 (13)	<0.001
Overall average presence^4^, hrs	16 (6)	5 (2)	<0.001
Continuous presence^5^, n (%)	28 (93)	0 (0)	<0.001
**SSC**^6^ **first week**			
Total SSC, hrs	34 (12)	21 (11)	<0.001
Mother, hrs	21 (10)	12 (8)	<0.001
Father, hrs	13 (7)	8 (5)	0.001
**SSC**^6^ **average/day**			
Total SSC, hrs	6 (2)	4 (2)	<0.001
Mother, hrs	4 (2)	3 (2)	0.002
Father, hrs	2 (1)	1 (0.6)	0.041

^1^ Independent *t-test* or Pearson’s chi-square tests.

^2^ One couple in SFR unit missing information regarding education level.

^3^ Postmenstrual age.

^4^ Daily registrations from birth to the infant reach gestational age (GA) 34 postmenstrual age.

^5^ Present ≥12 hours.

^6^ SSC: skin-to skin contact.

**Fig 2:**
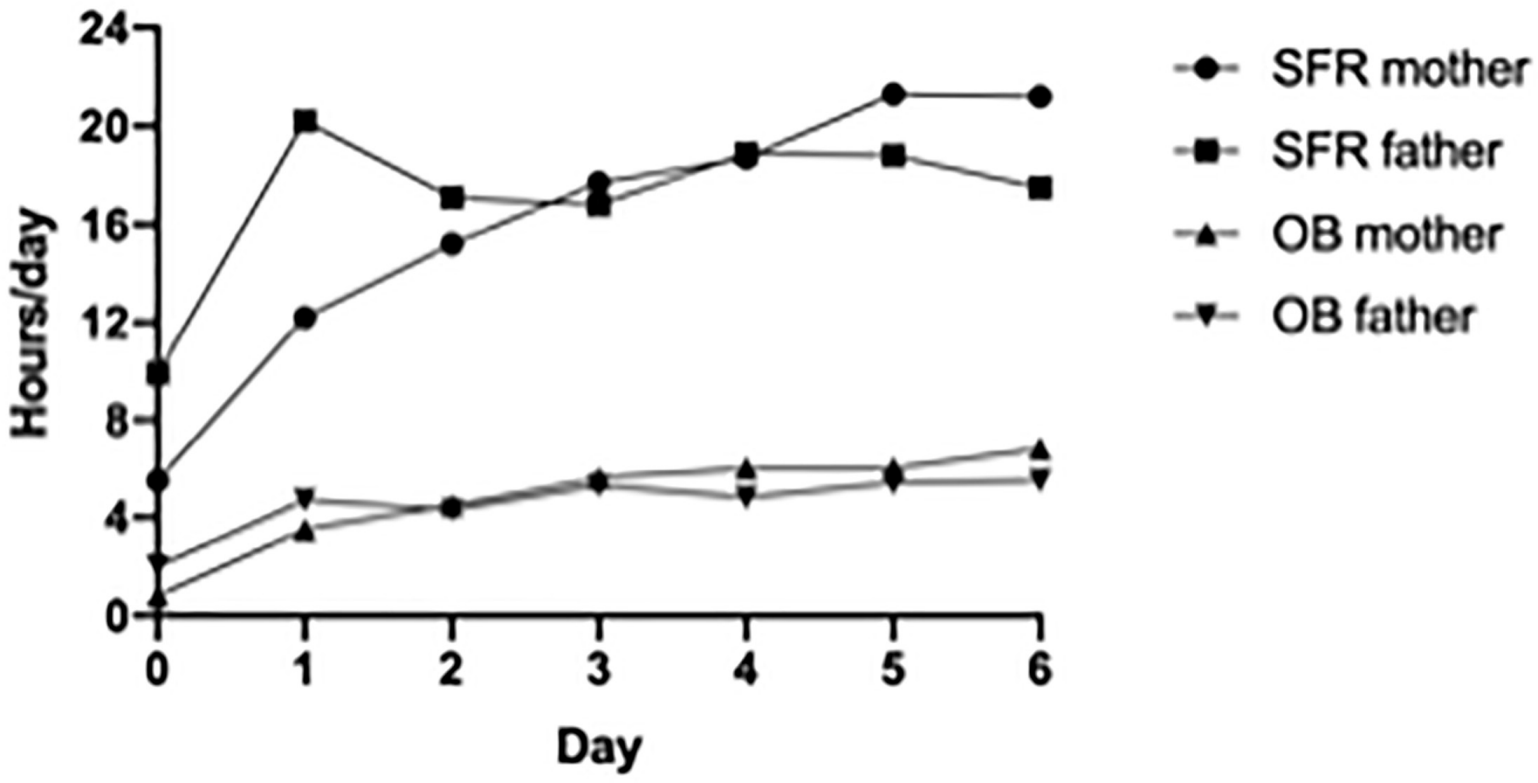
Parental presence first week. Mean hours of daily presence during first week of hospitalisation by mothers and fathers in the SFR unit and OB unit.

During the first week, both parents in the SFR unit were present for a mean of 20 hours per day, while the parents in the OB unit were present for a mean of four hours (Fig 2).

Eighty-seven percent of the mothers and 93% of the fathers in the SFR unit were continuously present (>12 hours per day), compared to none in the OB unit (Table 1). From birth until postmenstrual age of 34 weeks, parents in SFR maintained an average continued presence of 21 hours for mothers and 16 hours for fathers, compared, respectively, with seven hours and five hours in the OB unit. The respective mean daily hours of providing SSC from the second week were six and four hours (p<0.001) (Table 1).

The EPDS scores were lower for mothers in the SFR than in the OB unit from birth to four months corrected age, and the estimated difference for the period was -1.9 (95% CI: -3.6, -0.1) (Table 2). Table showing all covariates examined in the linear mixed model of repeated measurements are provided as supplement, S1 Table.

**Table 2:** Repeated measurements of depression, anxiety, stress and attachment by parents, presented as adjusted mean difference examined in linear mixed model.

	Mother				Father			
	Estimate*	SE	95% CI	*p-*value	Estimate*	SE	95% CI	*p*-value
**EPDS**								
Unit	-1.9	0.9	[-3.6, 0.1]	0.03	-0.5	0.9	[-2.3, 1.3]	0.58
**STAI**								
Unit	-3.0	2.3	[-7.7, 1.6]	0.20	-2.6	2.2	[-7.1, 1.9]	0.30
**PSS: NICU**								
Sights and sounds of the environment and Infant appearance								
Unit	-5.0	2.2	[-9.4, -0.6]	0.03	-5.3	2.1	[-9.5, -1.1]	0.01
Parental role alteration								
Unit	-5.2	1.8	[-8.7, -1.7]	0.004	-7.2	1.5	[-10.3,-4.2]	0.000
**PSI**								
Unit	2.8	4.5	[-6.2,11.8]	0.55	-0.5	5.4	[-11.2, 10.3]	0.93
**MPAS**								
Unit	-1.7	1.0	[-3.6, 0.3]	0.09	-0.5	1.2	[-3.0, 2.0]	0.68

* Estimate for the effect of unit, presented as adjusted mean difference between mothers and fathers, adjusted for mode of delivery (vaginal vs. caesarean section), parents’ education (elementary/high school or college/university) in linear mixed model: **EPDS** The Edinburgh Depression Scale. **STAI** The State–Trait–Anxiety Inventory, Short Form Y. **PSS: NICU** The Parent Stressor Scale: neonatal intensive care unit questionnaire. **PSI SF** The Parenting Stress Index—short form: Reporting the total stress score. All sub-scales within PSI were thoroughly checked. **MPAS** Maternal Postnatal Attachment Scale. Higher scores indicate more depression / more anxiety / more stress / more attachment

The difference between units was most pronounced during hospitalization, when 14% scored at a level indicating symptoms of depression in the SFR unit, as opposed to 52% in the OB unit (p<0.005) (Table 3).

**Table 3:** Mothers’ and fathers’ scores of depression, anxiety, stress and attachment, presented as means (SDs), median [Q1, Q3] or number (%) within each unit.

	SFR unit	OB unit	Difference between units*
AT DAY 14			*p-*value
**DEPRESSION**			
EPDS sum score, mothers	8 [6,11]	14 [10,15]	0.005
Depression symptoms (cut-off ≥13), mothers, n (%)	4 (14%)	16 (52%)	
EPDS sum score, fathers	6 [3,7]	8 [5,7]	0.17
Depression symptoms (cut-off ≥13), fathers, n (%)	1 (4%)	3 (11%)	
**ANXIETY**			
STAI-SF sum score, mothers	39 (13)	47 (13)	0.04
STAI-SF sum score, fathers	35 (10)	39 (14)	0.25
**STRESS, PPS: NICU**			
Sights and sounds of the environment and Infant appearance, mothers	35 (11)	39 (10)	0.12
Sights and sounds of the environment and Infant appearance, fathers	28 (10)	33 (9)	0.06
Parental role alteration, mothers	13 (7)	21 (8)	0.000
Parental role alteration, fathers	7 [4, 6]	12 [11,18]	0.003
**At DISCHARGE**			
**DEPRESSION**			
EPDS sum score, mothers	7 [5,10]	9 [7,10]	0.43
Depression symptoms (cut-off ≥13), mothers, n (%)	4 (15%)	3 (10%)	
EPDS sum score, fathers	4 [3,8]	6 [4,8]	0.57
Depression symptoms (cut-off ≥13), fathers n (%)	1 (4%)	4 (15%)	
**ANXIETY**			
STAI-SF sum score, mothers	37 (12)	34 (9)	0.48
STAI-SF sum score, fathers	32 (6)	31 (10)	0.73
**STRESS, PPS: NICU**			
Sights and sounds of the environment and Infant appearance, mothers	32 (14)	37 (12)	0.13
Sights and sounds of the environment and Infant appearance, fathers	25(9)	33 (11)	0.003
Parental role alteration, mothers	14[10,18]	17 [14,20]	0.06
Parental role alteration, fathers	7 [5, 9]	11 [10,15]	0.004
**BY TERM**			
**DEPRESSION**			
EPDS sum score, mothers	5 [3,6]	5 [4,7]	0.41
Depression symptoms (cut-off ≥13), mothers, n (%)	0 (0)	1 (3)	
EPDS sum score, fathers	3 [2,4]	3 [2,6]	0.24
Depression symptoms (cut-off ≥13), fathers, n (%)	0 (0)	1 (3)	
**ANXIETY**			
STAI-SF sum score, mothers	30 (9)	33 (11)	0.43
STAI-SF sum score, fathers	28 (8)	31 (10)	0.45
**STRESS, (PSI-SF)**			
Parental distress, mothers	19 [15,23]	23 [17,24]	0.29
Parental distress, fathers	21 [11,21]	19 [15,22]	0.68
Parent-child dysfunctional Interaction, mothers	20 [15,22]	19 [14,20]	0.71
Parent-child dysfunctional Interaction, fathers	17 (5)	17 (8)	0.16
Difficult child, mothers	18 (7)	19 (9)	0.75
Difficult child, fathers	17 [10,19]	21 [19,23]	0.15
Total stress, mothers	55 (25)	56 (26)	0.84
Total stress, fathers	59 [31,67]	58 [44,66]	0.38
**ATTACHMENT**			
MPAS sum score, mothers	92 (6)	95 (1)	0.05
MPAS sum score, fathers	77 (26)	88 (4)	0.10
**At 4th MONTH CORRECTED AGE**			
**DEPRESSION**			
EPDS sum score, mothers	4 [3,7]	5 [4,7]	0.65
Depression symptoms (cut-off ≥13), mothers, n (%)	1 (3)	1 (3)	
EPDS sum score, fathers	3 [2, 4]	3 [2,5]	0.92
Depression symptoms (cut-off ≥13), fathers, n (%)	0 (0)	1 (3)	
**ANXIETY**			
STAI-SF sum score, mothers	32 (11)	32 (8)	0.54
STAI-SF sum score, fathers	28 (7)	32 (9)	0.11
**STRESS, (PSI-SF)**			
Parental distress, mothers	20 (5)	19 (10)	0.60
Parental distress, fathers	19 (7)	17 (11)	0.50
Parent-child dysfunctional interaction, mothers	16 (4)	15 (8)	0.62
Parent-child dysfunctional interaction, fathers	16 (6)	15 (9)	0.16
Difficult child, mothers	19 (5)	17 (8)	0.23
Difficult child, fathers	20 (7)	19 (11)	0.77
Total stress, mothers	55 (18)	50 (25)	0.42
Total stress, fathers	55 (18)	51 (29)	0.68
**Attachment**			
MPAS sum score, mothers’	89 (3)	88 (8)	0.51
MPAS sum score, fathers’	85 (5)	84 (5)	0.49

**EPDS** The Edinburgh Depression Scale. **STAI** The State–Trait–Anxiety Inventory, Short Form Y. **PSS: NICU** The Parent Stressor Scale: neonatal intensive care unit questionnaire. **PSI** The parenting Stress Index—short form. **MPAS** Maternal Postnatal Attachment Scale

* Independent *t-test*.

During hospitalization, the SFR parents scored 8 points lower on the STAI-SF questionnaire (Table 3). Parents’ scores decreased to levels considered normal in both units at discharge and in the mixed model there was no significant difference between the units (Table 2).

Both SFR parents scored lower on stress related to “Sights and sounds of the environment” and “Infant’s appearance”, a mean difference of -5.0 (95% CI: -9.4, -0.6) by mothers and -5.3 (95% CI: -9.5, -1.1) by fathers. Also in regard to stress related to “Parental role alteration”, the SFR parents scored lower, with a mean difference of -5.2 (95% CI: -8.7, -1.6) by mothers and -7.2 (95% CI: -10.3, -4.2) reported by fathers.

From term date, there were no differences in PSI-SF scores between the groups in any of the sub-scales (Table 2). Parents in both units reported average scores just above the high-risk level (score of 52–90) and total stress scores remained in the lower part of the range defined as high-risk level (Table 3).

There were no significant differences between the units on the MPAS sum scores (Table 2). Mothers and fathers in both units scored high on parental attachment (Table 3).

## Discussion

To our knowledge, this is the first controlled study of emotional distress in a setting with documented continuous parental presence during their infant’s medical care. Emotional distress did not increase, and the risk of depression and stress were actually decreased among parents in the SFR unit compared to the OB unit. Of particular interest is the extensive presence by fathers in the SFR unit from the day of birth. Such extensive active participation throughout the infants’ stay by fathers has not previously been reported. Studies of fathers’ role and involvement in care and their contribution to the social–emotional development of preterm infants has just started to emerge [41, 42]; however, knowledge of *how* fathers increased involvement contributes to and affects the family is still limited [43].

Emotional distress was measured with five different questionnaires covering different aspects of emotional reactions and disturbance in attachment. We consider the consistency in the finding of no increased emotional distress in the SFR compared to the OB unit across the panel of questionnaires to strengthen our conclusion. Also, the uniform selection of patients, the similar right to health care services and parental leave, the clearly defined differences in design and the large differences in time parents were present in the two units strengthens the validity of the conclusion. The units were located more than 400 km apart, and they did not cooperate beyond this specific project. Therefore, a spill-over effect or negative expectations among participants, which may be a major challenge in true randomized controlled trials, seems rather unlikely [44].

We have previously shown that the study populations did not differ significantly in terms of morbidity and practices related to treatment and nutrition [24]. Although both units were the only units providing care for the eligible infants in their respective geographical areas and provided the same medical and nursing care, we cannot exclude unrecognized confounders related to care culture and practices. The OB unit also provides care for smaller and sicker infants, which could increase the general level of a stress in the unit. Studies using a quasi-randomization [20] or a before-and-after design with asynchrony in time between the study groups [15, 16] are also prone to the same and other confounders.

Lester et al. found that the effects of SFR were largely mediated through increased maternal involvement, breastfeeding and developmental care in the SFR unit. Optimizing facilities for parents of preterm infants in the NICU and thereby increasing parental presence and involvement may contribute to improved long-term outcomes [45]. Parents may provide unique sensory stimulation to their infants through SSC [46], talk and singing [47, 48]. The possibilities of such positive stimulation are better when parents are present around the clock compared to a few hours of visiting each day.

In our study, gestational age was higher and morbidity lower than in other studies reporting effects of SFR design [16, 49]. This may influence both the levels of distress and the extent of parental presence. In our experience, parents do not disappear or back out when the infant’s condition is deteriorating. Unfortunately we do not have data on maternal health (e.g., pre-eclampsia) before preterm delivery or about the parents’ previous mental health status. Both factors could potentially have some impact on the outcome measures, but there is no obvious reason why this should differ between the two study groups.

Regarding the difference in depression scores in mothers it is difficult to state a clinically relevant effect size precisely, but it has been proposed to be around four points [50]. Our results showed a difference of six points at day 14. It is relevant to speculate about an association between time spent per day by mothers with their infant and the risk of developing depressive symptoms. In the SFR unit, mothers were present daily three times longer than in the OB unit (21 vs. 7 hours). From a biological and evolutionary perspective, not being allowed or able to protect and take maternal responsibility for the infant would be expected to cause emotional distress and may explain the report of more depressive symptoms (52%) by mothers in the OB unit. However, only 6% of the fathers in the OB unit scored above the cut-off of >13 points at day 14, indicating a difference in vulnerability between mothers and fathers immediately after preterm birth.

Others have documented an increased burden of emotional distress on fathers after preterm birth [51, 52]. The extensive presence of fathers in SFR’s throughout the stay, with an average of 20 hours daily for the first three days, may provide additional emotional support for mothers who have been initially incapacitated and recovering from complications of pregnancy. How fathers’ biological emotional responses are programmed and developed towards their preterm infant has not yet been sufficiently explored [53]. We found that fathers did not report depressive symptoms, and this finding was similar in both units.

Both groups scored in the lower range for anxiety, indicating that this was not a predominant symptom among parents in any of these units. Infants included in the study carried a low risk for both short- and long term severe adverse outcomes, and this may have contributed to the low scores on anxiety.

Although stress among NICU parents is well documented by others [23, 54, 55], we found parents’ average stress scores to be in the lower range. Parents of preterm infants are undoubtedly prone to stress, but the effect sizes are small in populations with low morbidity and higher gestational ages [56]. Nevertheless, the differences in stress scores between the units were significant during hospitalization. The mean stress scores were more than five points higher for the mothers, and seven points for fathers in OB unit compared to the SFR unit, which could be considered clinically relevant. A previous study of the two units found that parents in the SFR unit gave higher scores on emotional support and participation [57]. Increased satisfaction with care may not necessarily decrease emotional distress, but a possible causality between the two deserves further research.

Pineda et al found slightly increased stress in mothers of infants hospitalized in single rooms and argued that stress was related to isolation, lack of support from other mothers, in addition to an increased feeling of obligation and responsibility of the infant. However the authors also hypothesised that the large variation in visitation could be associated with other factors like socioeconomic status and maternal health, and that they may have a larger impact on maternal stress than time present in a single-room [18].

In the SFR unit parents were included in daily rounds and may therefore represent the best continuity in the care of their infant [57]. When parents are involved and allowed unrestricted access, they participate actively in shared decision making at an informed and competent level, based on their knowledge of the infant. Most parents in the SFR unit are present also during night-time. They rarely leave the infant to the staff, and their continuous presence allows them to provide closeness and care immediately at the cues of the infant. This may reinforce parents’ feeling of being in control and provide stress relief. Aagaard et al. found mothering of a preterm infant to be a developmental process nurtured by close relationship with the infant [54]. The ability to be close to the infant is indeed enhanced in the SFR unit, and this may trigger positive emotions [58]. The questionnaire, *PSS*: *NICU*, may also predict depressive symptoms, and as such confirms the differences between the units from the EPDS scores. Still, at term date and at four months post-term, parents in both units scored just above the lower limit for high risk on the PSI questionnaire, without any difference between the units. This could question the validity of the results of the PSS: NICU, but it may just as well reflect stress experienced during the transition from hospital to home. Using a modified version of the PSI questionnaire, Flacking et al. found, in accordance with our findings, no overall effect of co-care vs. no co-care on stress, but reported more stress on a sub-scale related to feelings of incompetence among the mothers as a result of being unable to provide co-care [59].

Preterm infants can, for obvious reasons, only express their distress indirectly, through behavioural signals and physiological instability [60]. The long-term negative effects of infant stress during NICU care are also starting to emerge through follow-up studies with impaired neurodevelopment and psychological outcomes [55, 61]. The majority of effective non-pharmacological interventions to reduce infant pain and distress require active parental participation [16, 62–65]. Provided with facilities supporting presence in the SFR unit, parents chose to be present for most of the day and night. We therefore document that extensive parental participation is possible without increasing parents’ emotional distress; indeed, it seems to be reduced by continuous presence. Most research on the effect of positive stimulation and parental presence in the NICU has been conducted in preterm infants, but the same basic psychological needs mediated through parental closeness are present also for severely ill infants born at term. Parental presence and their vulnerability to psychological distress may be influenced by external factors such as differences in health care financing, a social welfare system compensating presence economically as recognized also by others [18]. To rebuild a NICU into SFR facilities are costly, potentially there could be cheaper interventions (e.g., frequent psychological support) to increase parents’ psychological wellness. However, a large and increasing evidence based knowledge of the medical and psychological benefits of parent–infant closeness in the NICU may point at near-continuous parental presence as one of the most underestimated interventions available in NICU care. A society and health care system adopting a policy allowing continuous parental presence takes a major step towards the goal of providing care at the premises of the patient and in accordance with the highest medical, legal and ethical standards.

## Conclusion

This study shows that continuous presence of both parents of infants hospitalised in a SFR NICU can be achieved without increasing parental distress. In addition, the risk of depression and stress decreased during hospitalization with potential long-term positive effects on parental well-being. Providing a NICU design that enables parents to stay continuously may also be beneficial for long-term outcomes of the infants. A physical design of the NICU facilitating the implementation of evidence-based practice of parental presence and participation should therefore be considered superior to a design limiting these possibilities.

## Supporting information

S1 Picture Patient and parents area in the SFR unit. Picture showing the patient and parents area in a single family room at Drammen hospital, Vestre viken HT.
(JPG)

S2 Picture Patient area in the SFR unit. Picture showing the patient in a single family room at Drammen hospital, Vestre viken HT.
(JPG)

S3 Picture Illustration photo of patient and parent in the SFR unit. Illustration photo a patient (a doll) and a mother (an employee) in a single family room at Drammen hospital, Vestre viken HT. The medical device is mounted on flexible arms so we can move the infant into the parent area without disconnect from monitoring and possible ventilation support.
(JPG)

S1 Table Table showing all covariates examined in the linear mixed model. Table 2 Showing all covariates examined in the linear mixed model of repeated measurements of depression, anxiety, stress and attachment by parents, presented as adjusted mean difference.
(DOCX)

S1 File Protocol. Protocol for the study “Impact of Family Centered Care in Single Family Rooms on Preterm infants and their Parents—A prospective comparative study”.
(DOCX)

S2 File TREND Checklist. Transparent Reporting of Evaluations with Nonrandomized Designs—TREND statement checklist.
(PDF)

## Abbreviations

EPDS: The Edinburgh Postnatal Depression Scale
MPAS: Maternal Postnatal Attachment Scale
NICU: Neonatal intensive care unit
OB: Open bay
PSI: The Parenting Stress Index—short form
PSS NICU: The Parent Stressor Scale: neonatal intensive care unit questionnaire
SFR: Single-family room
SSC: Skin-to-skin contact
STAI: The State–Trait–Anxiety Inventory, Short Form Y
