# Supplementary figures and images for: Parent psychological wellbeing in a single-family room versus an open bay neonatal intensive care unit
Source: PLoS One. 2019 Nov 5;14(11):e0224488. doi: 10.1371/journal.pone.0224488 (PMC6830777; doi:10.1371/journal.pone.0224488)

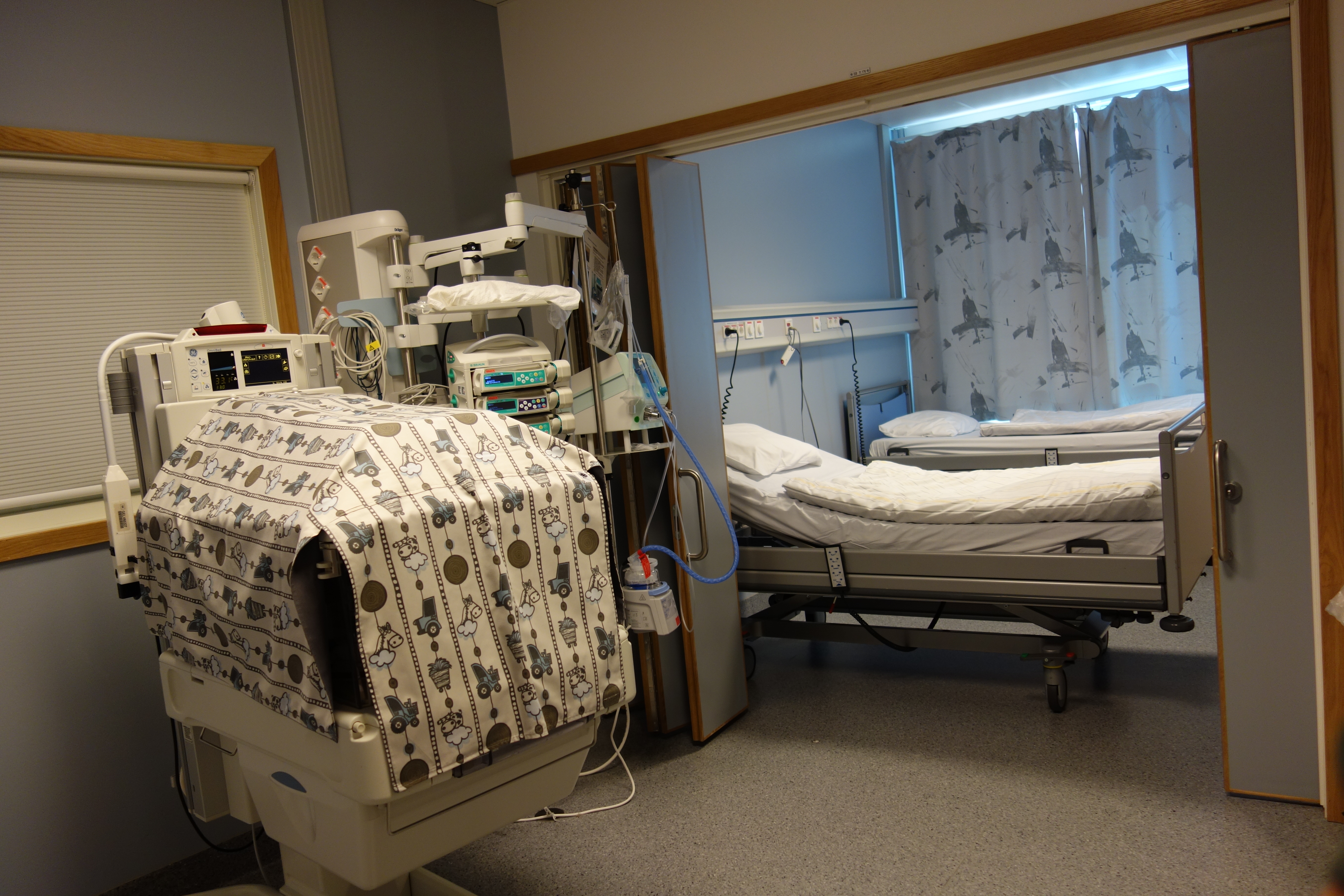

Supplement: S1 Picture — Picture showing the patient and parents area in a single family room at Drammen hospital, Vestre viken HT. (JPG) [file pone.0224488.s001.jpg]

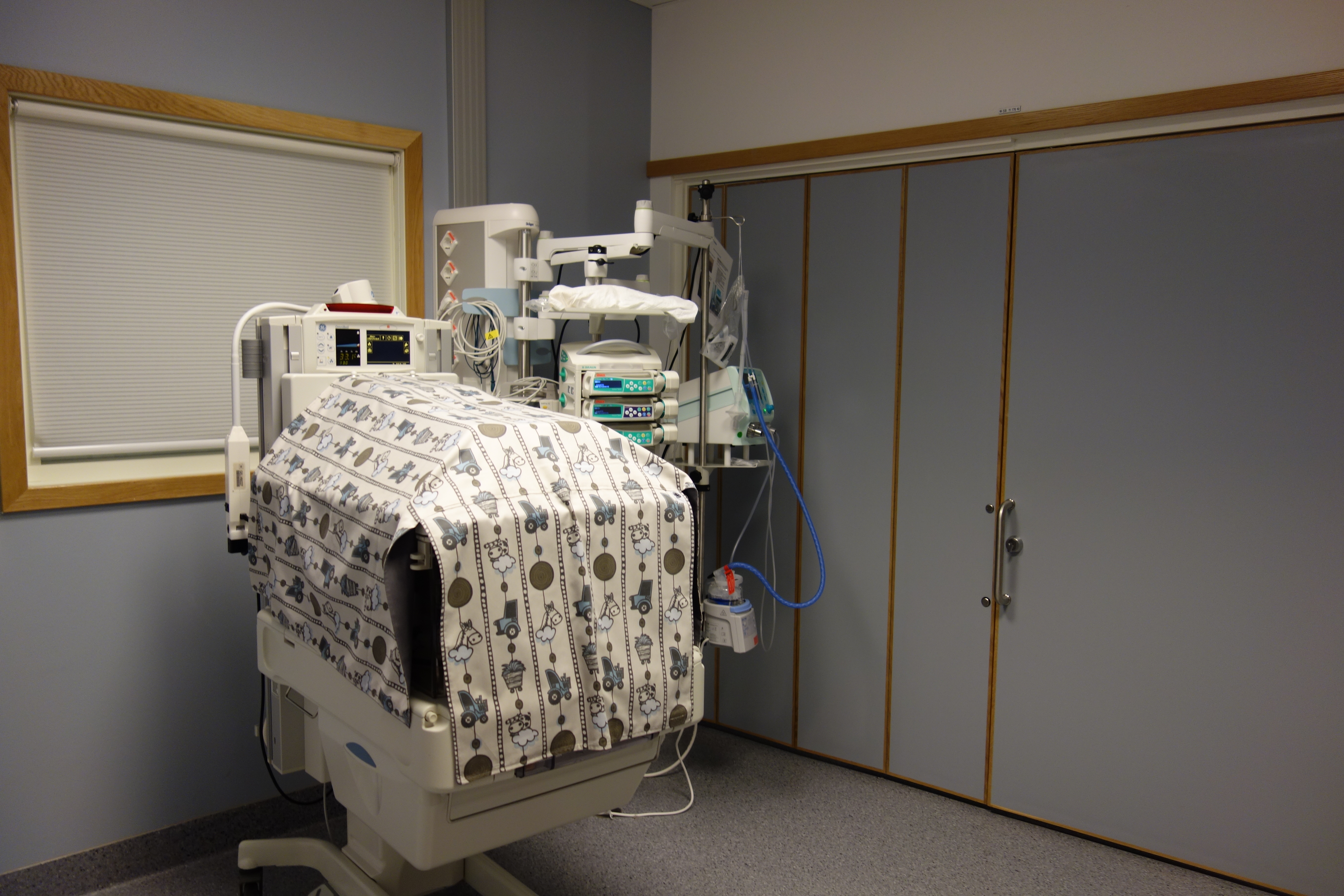

Supplement: S2 Picture — Picture showing the patient in a single family room at Drammen hospital, Vestre viken HT. (JPG) [file pone.0224488.s002.jpg]

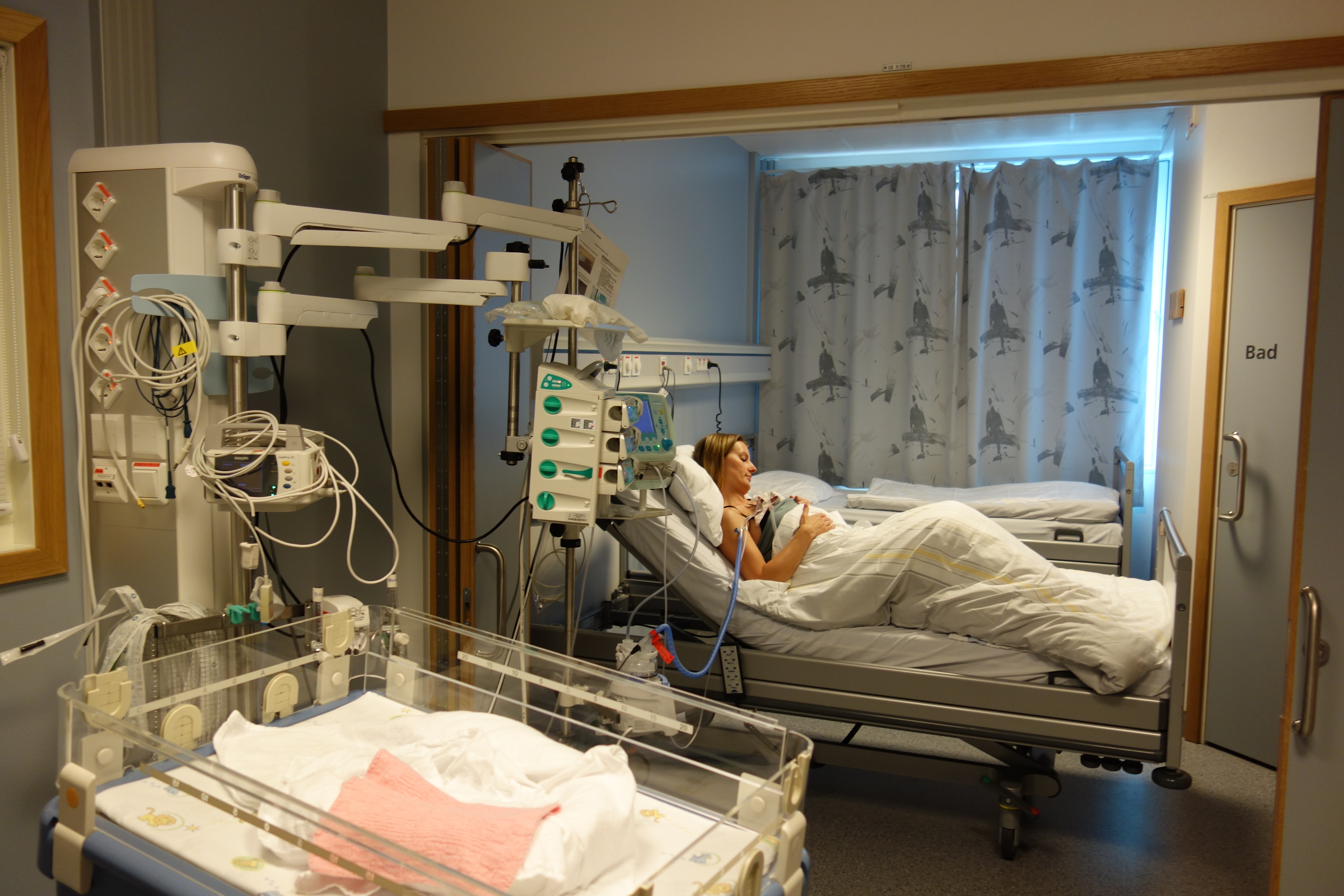

Supplement: S3 Picture — Illustration photo a patient (a doll) and a mother (an employee) in a single family room at Drammen hospital, Vestre viken HT. The medical device is mounted on flexible arms so we can move the infant into the parent area without disconnect from monitoring and possible ventilation support. (JPG) [file pone.0224488.s003.jpg]
