# Supplementary material for: Parent psychological wellbeing in a single-family room versus an open bay neonatal intensive care unit
Source: PLoS One. 2019 Nov 5;14(11):e0224488. doi: 10.1371/journal.pone.0224488 (PMC6830777; doi:10.1371/journal.pone.0224488)
Supplement: S1 Table — Table 2 Showing all covariates examined in the linear mixed model of repeated measurements of depression, anxiety, stress and attachment by parents, presented as adjusted mean difference. (DOCX) [file pone.0224488.s004.docx]

S4 Table

Table 2 Showing all covariates examined in the linear mixed model of repeated measurements of depression, anxiety, stress and attachment by parents, presented as adjusted mean difference.

| **MOTHER** | | | | | **FATHER** | | | |
| --- | --- | --- | --- | --- | --- | --- | --- | --- |
|  | **Estimate** | **SE** | **95% CI** | ***p-*value** | **Estimate** | **SE** | **95% CI** | ***p*-value** |
|  | | | | | | | | |
| **EPDS** |  |  |  |  |  |  |  |  |
| ^[[1]](#footnote-1)^Vaginal birth | -1.7 | 0.8 | [-3.4, -0.06] | 0.04 | 1.0 | 0.9 | [-2.7, 0.7] | 0.25 |
| ^[[2]](#footnote-2)^Education | 0.8 | 1.8 | [-2.8, 4.5] | 0.66 | 0.7 | 2.2 | [-5.0, 3.6] | 0.74 |
| ^[[3]](#footnote-3)^Time 2 | -2.1 | 0.6 | [-3.2, -0.9] | 0.000 | -0.1 | 0.5 | [-1.1, 0.9] | 0.86 |
| ^[[4]](#footnote-4)^Time 3 | -5.4 | 0.6 | [-6.6, -4.3] | 0.000 | -2.1 | 0.5 | [-3.1, -1.0] | 0.000 |
| ^[[5]](#footnote-5)^Time 4 | -5.1 | 0.6 | [-6.3, -3.9] | 0.000 | -2.8 | 0.5 | [-3.7, -1.7] | 0.000 |
|  |  |  |  |  |  |  |  |  |
| Unit | -1.9 | 0.9 | [-3.6, -0.1] | 0.03 | -0.5 | 0.9 | [-2.3, 1.3] | 0.58 |
|  |  |  |  |  |  |  |  |  |
| **STAI** |  |  |  |  |  |  |  |  |
| Vaginal birth | -0.1 | 2.2 | [-4.5, 4.4] | 0.98 | -2.3 | 2.2 | [-6.6, 2.1] | 0.30 |
| Education | 7.0 | 4.9 | [-2.7, 16.8] | 0.16 | 2.0 | 5.5 | [-9.1, 13.0] | 0.72 |
| Time 2 | -5.8 | 1.8 | [-9.3, -2.3] | 0.000 | -4.8 | 1.5 | [-7.1, 1.9] | 0.002 |
| Time 3 | -12.2 | 1.8 | [-15.8, -8.6] | 0.000 | -7.8 | 1.5 | [-10.9, -4.8] | 0.000 |
| Time 4 | -11.8 | 1.8 | [-15.4, -8.3] | 0.000 | - 7.13 | 1.5 | [-10.1, -4.1] | 0.000 |
|  |  |  |  |  |  |  |  |  |
| Unit | -3.0 | 2.3 | [-7.7, 1.6] | 0.20 | -2.6 | 2.2 | [-7.1, 1.9] | 0.30 |
|  |  |  |  |  |  |  |  |  |
| **PSS:NICU** |  |  |  |  |  |  |  |  |
| *Sights and sounds of the environment and Infant’s appearance* |  |  |  |  |  |  |  |  |
| Vaginal birth |  |  |  |  |  |  |  |  |
| Education | -0.4 | 2.1 | [-4.6, 3.7] | 0.85 | -0.4 | 2.0 | [-4.2, 3.7] | 0.88 |
| Time 2 | 04.apr | 4.5 | [-4.6, 13.4] | 0.34 | -4.4 | 4.9 | [-14.2, 5.3] | 0.37 |
|  | -1.8 | 2.1 | [-6.0, 2.3] | 0.39 | -1.2 | 1.9 | [-5.1, 2.6] | 0.52 |
| Unit |  |  |  |  |  |  |  |  |
|  | -5.0 | 2.2 | [-9.4, -0.6] | 0.03 | -5.3 | 2.1 | [-9.5, -1.1] | 0.01 |
|  |  |  |  |  |  |  |  |  |
| *Parental role alteration* |  |  |  |  |  |  |  |  |
| Vaginal birth | -0.5 | 1.6 | [-3.7, 2.7] | 0.76 | -0.2 | 1.5 | [-3.1, 2.7] | 0.88 |
| Education | -2.4 | 3.5 | [-9.3,4.5] | 0.49 | -4.2 | 3.6 | [-11.4, 2.8] | 0.24 |
| Time 2 | -0.8 | 1.6 | [-4.0,2.4] | 0.61 | 1.4 | 1.4 | [-1.4, 4.3] | 0.33 |
|  |  |  |  |  |  |  |  |  |
| Unit | -5.2 | 1.8 | [-8.7, -1.7] | 0.004 | -7.2 | 1.5 | [-10.3,-4.2] | 0.000 |
|  |  |  |  |  |  |  |  |  |
| **PSI** |  |  |  |  |  |  |  |  |
| Vaginal birth | 5.1 | 4.3 | [-3.5, 13.7] | 0.25 | 6.3 | 5.2 | [-4.0, 16.7] | 0.23 |
| Education | 3.1 | 10.0 | [-16.8, 22.9] | 0.76 | -17.3 | 14.3 | [-45.8, 11.2] | 0.23 |
| Time 4 | -3.6 | 4.4 | [-12.3, 5.2] | 0.42 | 2.4 | 5.2 | [-7.9, 12.7] | 0.65 |
|  |  |  |  |  |  |  |  |  |
| Unit | 2.8 | 4.5 | [-6.2, 11.8] | 0.55 | -0.5 | 5.4 | [-11.2, 10.3] | 0.93 |
|  |  |  |  |  |  |  |  |  |
| **MPAS** |  |  |  |  |  |  |  |  |
| Vaginal birth | -1.3 | 1.0 | [-3.2,0.6] | 0.18 | 0.4 | 1.2 | [-2.1, 2.7] | 0.78 |
| Education | 1.4 | 1.7 | [-2.0, 4.8] | 0.41 | -14.5 | 4.0 | [-22.6, -6.5] | 0.001 |
| Time 4 | -4.8 | 1.0 | [-6.9,-2.8] | 0.000 | -0.2 | 1.7 | [-3.7, 3.1] | 0.90 |
|  |  |  |  |  |  |  |  |  |
| Unit | -1.7 | 1.0 | [-3.6, 0.3] | 0.09 | -0.5 | 1.2 | [-3.0, 2.0] | 0.68 |
|  |  |  |  |  |  |  |  |  |
|  |  |  |  |  |  |  |  |  |

**EPDS** The Edinburgh Depression Scale. **STAI** The State–Trait–Anxiety Inventory, Short Form Y. **PSS: NICU** The Parent Stressor Scale: neonatal intensive care unit questionnaire. **PSI** The Parenting Stress Index – short form. Reporting the total stress score. All sub-scales within PSI were thoroughly checked, and there were no differences between the units or between genders.

**MPAS** Maternal Postnatal Attachment Scale.

Vaginal vs. caesarean section.

Elementary, high school or college/university.

Time 2 Discharge.

Time 3 Term age.

Time 4 Four months after term age.

1. [↑](#footnote-ref-1)
2. [↑](#footnote-ref-2)
3. [↑](#footnote-ref-3)
4. [↑](#footnote-ref-4)
5. [↑](#footnote-ref-5)
