# Supplementary material for: Parent psychological wellbeing in a single-family room versus an open bay neonatal intensive care unit
Source: PLoS One. 2019 Nov 5;14(11):e0224488. doi: 10.1371/journal.pone.0224488 (PMC6830777; doi:10.1371/journal.pone.0224488)
Supplement: S1 File — Protocol for the study “Impact of Family Centered Care in Single Family Rooms on Preterm infants and their Parents—A prospective comparative study”. (DOCX) [file pone.0224488.s005.docx]

**Impact of Family Centered Care in Single Family Rooms on Preterm infants and their Parents**

**A prospective comparative study**

1. **Introduction**

Over the last decades, care for prematurely born infants in the neonatal intensive care unit (NICU) has changed dramatically. Earlier, naked babies were exposed to bright light and high levels of noise in noisy incubators placed in rooms similar to operating theaters. In addition, they were frequently disturbed for intrusive procedures, e.g. for weighing, other measurements and blood tests. With the invention of improved treatment modalities and, in particular, monitoring technology, e.g. for monitoring ventilation and oxygenation, the need for direct visual observation has been reduced to a minimum. This has allowed for shielding the infant from light and noise, much less handling, and for safely taking infants out of incubators to be handled by parents, in particular allowing prolonged periods of skin-to-skin contact (SSC) with parents. Currently, a new paradigm of care has been suggested, i.e. family centered care (FCC) in single rooms. Historically, the FCC probably had its origin back in the 1950s, when scientist Bowlby and missionary Johnson both worked in hospitals in London ([1](#_ENREF_1)). Their experiences of separation of mother and child led to Bowlbys recognized theories of the benefit of attachment as we know them today ([2](#_ENREF_2)). FCC has been developed further, and today we define it as: “a way of caring for children and their families within health services which ensures that care is planned around the whole family, not just the individual child/person, and in which all the family members are recognized as care recipients” ([3](#_ENREF_3)). The philosophy is that parents take a more central role in the care of the infant in the NICU while the nurse is assisting the parents; e.g.it includes single family rooms (SFR) and possibility for parents to be present 24 hours a day. Favorable experiences have been reported, but they are few, are hampered by short experiences and short follow-ups by people with an invested interest, and the studies have, in our opinion, low scientific merit. Indeed, a recent scientific study published in a major journal raised concern that single room management of infants may have an untoward effect on development during the first two years of life ([4](#_ENREF_4)). However, it is important to emphasize that parents’ presence in that study is not necessarily comparable to Nordic countries; in Nordic countries parents of hospitalized infants have more extensive social rights. The project scope is examining if involvement of parents in their infant’s care and treatment affects the infants and the parents themselves. Practically, this project examines the effects of user participation.

This study is a joint venture between Haukeland University Hospital (HUH) and Vestre Viken Hospital Trust (VVHT). The study compares traditional care and FCC in NICU in SFR. The NICU at HUH was built around 1980 as a traditional Open bay (OB) unit. It is crowded, has no single rooms or access to rooming in with the parents. The NICU at VVHT opened in April 2012 and it is unique in its kind in a Norwegian context. There are similar units in Uppsala and in Stockholm, but VVHT is the first to provide SFR facilities also during intensive care treatment 24/7. FCC has been increasingly emphasized as an important and necessary element of neonatal intensive care ([7](#_ENREF_7)). A shift from traditional NICU to a FCC unit involves both physical structural changes and involves the entire interdisciplinary team around the individual families. The main difference is firstly the involvement of parents and their right to be present together with infant and secondly the establishment of parents as the responsible caregivers for the infant, even when the infant is hospitalized.

- 1. **Previous research**

**Effects of Single Family Room Care (SFR)**

A recent review ([8](#_ENREF_8)) assessed what benefits SFR NICU design may have compared to an OB unit environment in terms of privacy; increased parental involvement in patient care, infection control, noise control, improved sleep and decreased length of stay, and that such factors need to be considered by health service planners when designing NICUs ([8](#_ENREF_8)). A randomized controlled trial from Stockholm showed that the implementation of FCC and presence of parents in the unit for 24 hours/day from admission to discharge, reduced the overall length of stay for premature infants (5.3 days) ([9](#_ENREF_9)). The risk of moderate-to-severe bronchopulmonary dysplasia (BPD) was also decreased in the FCC group. A study from Washington reported increased parental visitation during the first four weeks in a SFR NICU compared with an OB NICU, but also more stress among mothers in the SFR unit ([10](#_ENREF_10)). However, the studies in their designs were subject to confounding, and the Stockholm study basically involved moderately preterm infants not in intensive care (Level II nursery). Furthermore, the studies only provided meaningful information on very limited outcomes, i.e. length of stay and maternal stress, and the results of the intervention were partly opposite in the two studies. The studies did not address the most important questions related to differences in care, e.g. whether degree of parental presence in the NICU was of significance for neonatal morbidity, growth, successful attainment of breastfeeding or parental coping at or beyond discharge, except that the one study actually reported *increased* parental stress with increased NICU presence.

**Kangaroo Mother Care (KMC) and skin-to-skin contact (SSC)**

KMC is defined as: “ Early, prolonged, and continues skin-to-skin contact between a mother and her newborn low birth-weight infant (<2500g, viz preterm and or low birth weight infant), both in hospital and after early discharge, with (ideally) exclusive breastfeeding and proper follow –up”([11](#_ENREF_11)). A Cochrane review concluded that SSC was associated with reduction in mortality, severe infections, hypothermia and severe illness, lower respiratory tract disease, shorter length of hospital stay, increased weight gain and growth of head circumference and length, increased breastfeeding rate and maternal satisfaction ([12](#_ENREF_12)). SSC may also reduce autonomic pain responses, and have positive effects on infants sleep patterns and on psychological aspects like improved parent – infant interactions in preterm infant ([13](#_ENREF_13)). SSC may also help fathers to attain their paternal role and to cope with the unexpected situation ([14](#_ENREF_14)). In another review it is argued that SSC may be a parental activity that enhances parental attachment and nurturing the infant – and thereby promotes better growth ([15](#_ENREF_15)). The benefits of KMC are mainly seen in low income countries, and objective clinical significance in high income countries like Norway, is not established. As of today, no one can point out exactly how many hours of SSC are necessary to obtain positive results. SSC is provided in the NICUs both at HUH and VVHT, but due to facility restraints, more extensive SSC can be practiced at VVHT. There is a lack of systematic knowledge about how many hours SSC it is possible to achieve in a FCC-unit, and if there is an association between extent of SSC and possible benefits or untoward effects.

**Nutrition, growth and breastfeeding for premature**

There is general agreement that one aim of care in the NICU is to obtain a growth in weight, body length and head circumference close to expected intrauterine growth although the optimal timing of catch-up growth is debated. Above all, there is consensus that breast milk is the preferred enteral nutrition ([16](#_ENREF_16)). There are, however, diversity of practice in how to go about nutrition, e.g. with respect to fortifying breast milk and what is considered appropriate volumes, which may represent a challenge for designing studies on growth in preterm infants. In order to measure the effect of traditional vs. FCC unit care, nutrition protocol is standardized by the present comparison study between HUH and VVHT. Nutritional needs and energy requirements may be affected by the infant's total energy expenditure. Stressful environment and procedures may increases energy expenditure required for adequate growth, and eliminating sources of stress may increase growth due to more energy available for growth. The categorization of breastfeeding will follow the World Health Organization’s criteria that define selected infant feeding practices, distinguishing between four categories ([17](#_ENREF_17)).

**Parental health**

Parental stress may be caused by a variety of factors, including mismatch between the perceived demands of parenting and the resources available to meet those demands ([18](#_ENREF_18)). Furthermore, it is well documented that mothers perceive stress after delivering a premature infant ([19-21](#_ENREF_19)) and that preterm birth increases the risk for postpartum depression ([22](#_ENREF_22)). Some studies suggest that fathers are also prone to stress ([23-26](#_ENREF_23)), but may have a different path of stress than mothers ([26-28](#_ENREF_26)). Providing guidance to parents in observing their infant’s behavior and development has improved parent-infant interaction and reduced maternal anxiety and depression ([29](#_ENREF_29), [30](#_ENREF_30)). In a SFR unit both parents may become earlier and more directly involved in the overall care of their premature infant, and we hypothesize that this may promote sharing of responsibility by the parents and make both parents more confident and better prepared to share responsibility, interact with and care for their infant both at discharge and later at home. This hypothesis needs to be tested since it is also a possibility that the effect may be the opposite since extended presence in the NICU may lead to feeling of excessive pressure to take responsibility and to more problems related to other children and social network.

**Nurse-parent perspectives to parent support/participation**

Potentially, nurses may promote mutual decision making and empathetic communication with parents. It would be important to ask both parents and nurses about the level of support parents are given. This information would help us to understand if parent support is appropriate to enable their active participation in infant care and decision making.

- 1. **The potential impact of the project**

The current study consists of two parts; the main part of the study, **The Comparison Study** examines outcome in relation to the health of the premature infant and their parents in a FCC unit with SFR compared to a corresponding group in traditional OB NICU unit. There is a major renovation and establishment of new buildings at Norwegian hospitals. The study could be of importance in accordance with the recommendation of guidelines and the construction of future NICU nationally and internationally.

A second minor part of the study is The International Closeness Survey study (ICS) conducted by the international SCENE research group. The aim of **the ICS Study** ([5](#_ENREF_5), [6](#_ENREF_6)) is to study if, and if any, how the culture of neonatal units across Europe promote or is a hindrance for parent-infant closeness (physical and emotional) and what the psychological, social and biological effects of closeness are. Two Norwegian neonatal intensive care units (NICU) participated in this study; HUH and VVHT. The ICS study has been important in identifying similarities and differences between the two units who will take part in The Comparison study. It is particularly important that HUH and VVHF participated because the study will describe an overall difference/similarity between the two NICUs with regard to family participation in care and extent of skin-to-skin contact. Such a base registration is of paramount importance to interpret the results of the comparison study.

1. **Aims**

We have the following objectives with the study (of which the italics have been completed):

**The Comparison Study**

1. *Could Family Centered Care (FCC) with Skin-to-skin contact (SSC) in Single Family Room Care (SFR) affect the preterm`s growth (gain in weight, length and head circumference)* *while in NICU and during the first 4 months’ corrected age (age from expected term date)?*
2. *What is the effect of FCC in SFR vs traditional open bay NICU care on the infant in terms of length of stay in the NICU and neonatal morbidity; iatrogenic complications to treatment, (infections, lung disease, cerebral bleeds when potential confounders (gestational age, birth weight, family’s sociodemographic characteristics) are accounted for.*
3. Does SFR design have impact on parents’ emotional and social wellbeing or stress and competence in caring for the infant, during the NICU stay and after during the first 4 months’ corrected age?
4. What is the effect of FCC in SFR vs traditional open bay NICU care on breastfeeding rates, provision of mother’s milk and mothers’ confidence in breastfeeding?

**ICS Study**

1. *Variations in attitude and accomplishment of parental presence and SSC in Europe,*
2. *How different organizations in this respect influence parent satisfaction and participation and nurses’ experience of support with the parents?*

**Hypotheses**

The null hypotheses are that SFR design does not impact on preterm infants’ health (growth and morbidity) and their parents psychosocial wellbeing or breast feeding compared to traditional OB NICU design.

1. **Study population and Methods**

**The Comparison study between VVHF and HUH**

The Cochrane study which argued for an urgent need to scientifically assess the effect of FCC ([3](#_ENREF_3)) also discussed methodological challenges and concluded that a randomized controlled study (RCT) in a single unit would be ethically and methodologically impossible to conduct, e.g. since it will be impossible to avoid “contamination” between groups, and argued that comparison between different units would be the most valid method. This recommended approach will be followed in the present study, and the challenge is to minimize factors that may act as confounders. To minimize confounding the following details on design will be adhered to:

**Recruitment of comparable infant - parent dyads,** limitation to infants with gestational age 28-32 weeks and exclude infants with malformations which need special care and treatment. **Strict definitions of exposure variables,** in addition to elements related to different NICU setting; **(1)** *nutrition,* e.g. in terms of type of nutrition (parenteral, mother’s milk, banked breast milk), mode of increment until full enteral feeds, type and timing of fortification of breast-milk, volume of milk per day based on body weight. (2) *parental presence and duration of SSC.*

**Strict definitions and prospective registration of outcome variables;** (1) *growth*; weight, body length and head circumference at 34 postmenstrual age (PMA), and at discharge; methods and timing of recording weight, length and head circumference and recording of gain in growth PMA at discharge will be recorded. (2) *treatment procedures* (ventilation support, blood sampling etc) uniform monitoring routines, *mobidity* according to specific definition i.g sepsis)**.** (3) *parents experience* of psychosocial stress and confidence in caring for their infants will be outcomes reported by questioners’ at four different time point (at day 14.th, at discharge, by term and by fourth month corrected age). (4) *rate of breastfeeding* and mothers’ confidence in breastfeeding at discharge and postmenstrual age (PMA) when accomplishing breast-feeding, After discharge from the hospital, data will be obtained on two occasions; at *term* and at *four month corrected age*. The parents will be requested to come for follow-up at the hospital for vital measurements rate of full and partial breast-feeding at term and 4 months’ corrected age of the infants.

**The questionnaires**

Parents will answer six different questioners’*:*

1. *The Parental stress scale: NICU (PSS:NICU)* will be assessed after two weeks of hospitalization and at discharge. The instrument was designed to determine parental perception of stressors arising from the physical and psychosocial environment in the NICU ([34](#_ENREF_34)). The PSS: NICU is used in another study in Norwegian ([27](#_ENREF_27)) but has not been validated in the Norwegian version. However, the tool has been validated for comparable European populations, and since this is a comparative study, national validation is of limited importance.
2. *The Edinburg Postnatal Depression Scale (EPDS)* will be tested after two weeks of hospitalization, at discharge, at tem and at 4 months’ corrected age. The instrument is developed by psychiatrist John Cox and his colleagues ([35](#_ENREF_35)). It is used extensively in research as well as a diagnostic tool. The EPDS is translated into more than 25 languages, including Norwegian and was validated for Norwegian condition by Eberhard-Gran and Berle ([36](#_ENREF_36), [37](#_ENREF_37)). EPDS consists of 10 statements, each has four response options.
3. *The STAI Short Form Y (STAI)* will be tested after two weeks of hospitalization, at discharge, at tem and at 4 months’ corrected age. STAI is an instrument for measuring anxiety in adults ([38](#_ENREF_38)). It differentiates between the temporary condition of “state anxiety” and the more general and long-standing quality of “trait anxiety”. STAI is one of the most frequently used measures of anxieties applied in psychology research ([39](#_ENREF_39)).
4. *“The Breastfeeding Self-Efficacy Scale-Short Form” (BSES-SF****)*** ([31](#_ENREF_31)) at discharge. This instrument addresses confidence in breastfeeding. A Norwegian version is published by Dr. Silje Haga ([32](#_ENREF_32)). This instrument is shown as a unique tool to identify breastfeeding mothers of preterm infants at risk of prematurely weaning ([33](#_ENREF_33)). Since this is a comparative study, national validation is of limited importance.
5. *Parenting Stress Index (PSI)*. The short form (36 questions) of PSI is a widely used clinical and research self-report instrument for assessing parent stress due to parental factors or deviant development of the child and for identifying risk of dysfunctional parenting ([41](#_ENREF_41), [42](#_ENREF_42)). A Norwegian version has been validated ([43](#_ENREF_43)). The questionnaire includes a parent domain and a child domain where the parent domain addresses social isolation, attachment to the child, health, role restriction, depression and partner, and the child domain includes distractibility / hyperactivity, adaptability, reinforcement of parent, how demanding the child is perceived to be, mood and acceptance. The two domains taken together make up the overall stress level ([44](#_ENREF_44)).
6. *Maternal Postnatal Attachment Scale (MPAS)*.This instrument will provide complementary information related to evaluation of the prevalence of stress/anxiety/depression. This in view of the possible high score of stress/anxiety/depression is associated with interacting with the infant. This instrument evaluates the parents’ own feelings regarding interaction with the infant([40](#_ENREF_40)). It will not provide sufficient detailed knowledge to be able to conclude on the quality of interaction of the individual participating families, but it will provide information regarding possible differences between groups. The instrument is available in a Swedish version.

**Inclusion and participants**

In *The Comparative study*, the number of eligible infants (gestational age 28-32 weeks without congenital malformations or chronic disease) per year is approximately 50 at VVHT and 55 from Hordaland county at HUH. The recruited mother-parent dyads will thus be population based, i.e. representing Buskerud and Hordaland. To assure comparable cohorts, we limited the study to infants born at 28^0^–32^0^ weeks’ gestation of families living in the respective catchment areas. Each unit was the only NICU in their respective areas. We excluded infants with congenital malformations, major complications, such as intraventricular hemorrhage grade III/ IV or surgically treated necrotizing enterocolitis, and infants with a BW less than 800 grams. We also excluded infants where one or both parent(s) had a major mental illness or did not understand Norwegian language, infants of mothers who had taken illicit drugs or were on methadone during pregnancy, and infants who had been in the custody of Child Protection Services from birth.


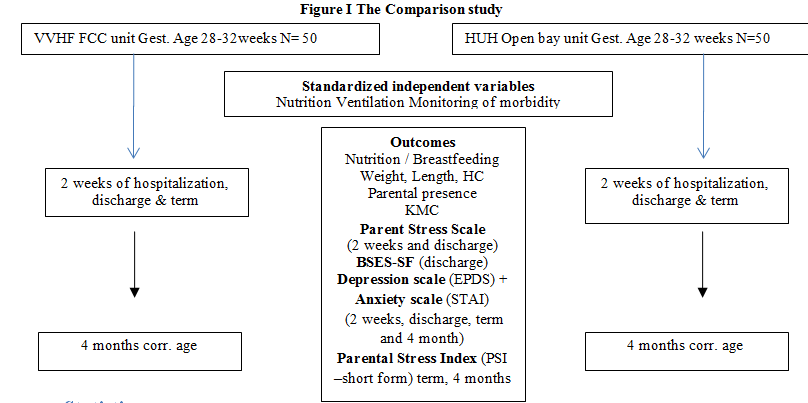


**Power calculations**

The study was powered to examine the difference in weight at discharge between the SFR and OB units. In the pilot study at VVHT, average weight at discharge was 300 grams higher in the SFR unit than in the OB unit. Based on an expected difference of 300 grams, a power calculation suggested that 10 infants were needed in each group to obtain a significant result with a p < 0.05 and a power of 80%. However, the observed difference occurred in parallel with the reorganization within the hospital and was not necessarily representative when comparing the two units. We therefore chose to include up to 80 infants. Based on admissions during previous years, the plan was to include 42 infants in each group. Inclusion started on May 1, 2014 and had to end on July 31, 2016 because the OB unit would be moved to another building. All patients have completed the study (April 2017).

**Statistics**

Data will be presented as descriptive statistic, measurements with two-sample *t*-tests and Pearson’s chi-square tests. Linear regression analysis will be used to estimate the mean difference between the SFR and OB after adjusting for potential confounders (parents’ education, for mode of delivery (caesarian section or vaginal delivery) and gestational age at birth). Mean differences in measures of growth (weight, length, and head circumference) from birth to four months after expected term date were analyzed with linear mixed models in cooperation with a biostatistician.

**The International Closeness Survey (the ICS Study)**

In the ICS study qualitative and quantitative methods are used to get a full picture of closeness-promoting culture and its effects on the infant, family and staff. Data have been collected prospectively to evaluate different NICU units in Europe. Every participating NICU will recruit at least 30 study patients The methods include a questionnaire which is used to collect background information of infant/parents and data for the secondary outcomes (growth, hospital stay) and about the unit. The time of parents’ presence at the NICU and daily amount of closeness (parent-infant skin-to-skin) are reported with Parental Closeness Diary. The extent of parent participation and the support parents receive from the staff is measured using 9 SMS questions covering aspects of staff support, e.g active listening and parent participation in infant care. These questions on staff support are sent in random order as SMS to the mobile phone of both parents. Similar questions (about provided support) corresponding to the parents’ questions, are also asked to the nurses, measured with Web-questions. All units recruit parents for 3 months and follow the recruited parents until infant discharge.

1. **Organization**

This project has been developed in collaboration with the NICU at HUS together with the current research leader Professor Trond Markestad. HUH has contributed to the recruitment of families for the control group as well as the funding of research nurse at HUS. Drammen Hospital and HUH has an agreement that PhD credits’ points accrues to Drammen Hospital. The NICU at Drammen HT was invited in to the European Research Group SCENE (Separation and Closeness Experiences in the Neonatal Environment) in 2013. SCENE is a multi-disciplinary group of international professionals that aims to improve parents’ and infants’ experiences and outcomes of neonatal care. The group meets once every six months and carries out multinational studies providing knowledge about family-based care from a wide interdisciplinary and multi-methodological perspective. The candidate is in the steering group. Another member of the SCENE Group Steering Group, Professor Renèe Flacking, is directly involved in the project as a supervisor. Cooperation with the group and prestigious international researchers through participation in international studies is of great importance for the NICU at Drammen Hospital. Before this study, Child and adolescence department had little research experience and affiliation - but now the collaboration is well established and the Head of the department, Krzysztof Hochnowski, have been linked to the SCENE group.

Both the NICU at Drammen Hospital, VVHF and the candidate have a close cooperation with the Norwegian Association “Prematurforeningen”.

| **Affirmation/Organization** | **Name** | **Role in the project** | **Competance** |
| --- | --- | --- | --- |
| Drammen Hospital/ Oslo University Hospital  University of Bergen  Dalarna University (Sweden) | Atle Moen  Trond Markestad  Renée Flacking | Supervisor, MD, PhD  Supervisor, Professor  Supervisor, Professor | Research, Neonatal medicine  Research, Neonatal medicine  Research, Neonatal nursing |
| Drammen Hospital | Bente Silnes Tandberg | PhD Candate,MNSc | Neonatal nursing, |
| Oslo University Hospital | Kathrin Frey Frøslie | Statistician advisor, PhD | Biostatistics’ |
| Haukeland University Hospital, NICU  Prematurforeningen | Hege Grundt  Hege Nordhus | Research nurse, master student  Head of the user organization, the Norwegian Association “Prematurforeningen” | Neonatal nursing |
| Drammen Hospital, VVHF HT, NICU | Kristoffer Hownoski | Head of the department | Neontal mediscine |
| Turku University Hospital/ Turku University | Liisa Lethonen | Head of The SCENE reseach group, and project leader for ICS Study, Professor, MD | Research, Neonatal medicine |

1. **Project plan**

The recruitment for The Comparison study started in May 2014 and was completed in July 2016. Two articles have been completed, article 3 will be completed before Christmas 2018 and the last article is scheduled to be written in 2019, together with the compilation. Parts of the results have already been presented internationally at several scientific congresses and further presentations are planned in 2018 and 2019. The results of the study will also be made known to the user organization (Premature Association) 30th anniversary in December 2018. The thesis submits to University of Bergen during summer 2019.

Inclusion of patients to the ICS study was completed in April 2014. The results are published.

1. **Ethics**

The parents received oral and written information and were included if both of them gave written consent within the second day. The study was approved by the Norwegian Regional Committee for Medical Research Ethics and registered in ClinicalTrials.Gov (NCT 02452580) and at www.kliniskestudier.no. Data is stored in a database containing name, personal identification number, a code number which corresponds to other files. The data will be stored at the respective Research servers at VVHT and HUH according to the requirements of the hospitals and REK (de-identified study file and a file with a key to the study file). Transfer of data from HUH to VVHF will be performed as encrypted files according to requirements. All participants will be asked to sign an informed consent form at the enrolment in the study. The nurses in the ICS study participated anonymously and the institution gave the consents. The communication with the SMS service provider is secured using SSL, which is the standard method for encrypted www traffic.

1. **Financing**

In the initial phase this study was supported by research grant from VVHT and HUS University hospital and The Norwegian Nurses Organization. The main phases of this study were supported by research grant from and the Norwegian Extra Foundation for Health and Rehabilitation for 2, 5 year. The candidate needs research grants for buy-out from a clinical position for 0.5 year to completion of PhD in 2019; statistic and writing of the last two articles as well as compilation of doctoral dissertation.

1. **Milestones**

The candidate has completed the PhD program at University of Bergen. The data collection was completed July 2016.

**Scientific presentations**

- The SCENE Symposium at several occasions; Turku, 2014, Drammen 2016, and too be presented at Como in 2018
- The European Academy for Pediatric Societies in Barcelona, October 2014
- The 31st Annual Graven Conference on the Environment of Care for High Risk Newborns, in collaboration with the March of Dimes, Florida, USA in March 2018
- The Nordic Conference in Nursing Research, Oslo, June 2018.

1. **Plans for publication**

The results will be presented at international and national scientific meetings and published in international peer-reviewed journals.

**The Comparison study**

Follows the Vancouver criteria’s and there is an agreement between the institutions that also deals with of authorship of publications. One article, *Impact of Single Family Room NICU care on pre-and post-discharge growth, parental presence, and skin-to-skin contact,* is submitted to Pediatrics (July 2018).

A second article, *Effect of single room FCC vs. OBC on parental experience of stress, anxiety, depression and attachment during care in the neonatal intensive care unit and the first 4 months after term date,* is initiated. Article 3*Effect of single room FCC vs. OBC on maternal breast-feeding confidence and rate of breastfeeding during care in the neonatal intensive care unit and the first 4 months after term in infants born very preterm* (a master study) are planned for submission during last quarter 2018 and spring 2019.

**The ICS study**

The authorship is set to follow the NEJM 1997 criteria for authorship + the journal-specific criteria for Scene steering group: On article is accepted June 2018 for publication in Journal of Perinatal and Neonatal Nursing; *Parent–Infant Closeness, Parents’ Participation, and Nursing Support in Single-Family Room and Open Bay NICUs*.

In addition the candidate is also co-author of two articles published by the SCENE research group.

Referencelist

1.Alsop‐Shields L, Mohay H. John Bowlby and James Robertson: theorists, scientists and crusaders for improvements in the care of children in hospital. Journal of Advanced Nursing. 2001;35(1):50-8.

2.Bowlby J. A secure base: Parent-child attachment and healthy human development: Basic Books; 2008.

3.Shields L, Pratt J, Davis LM, Hunter J. Family-centred care for children in hospital. Cochrane Database of Systematic Reviews. 2007;1.

4.Pineda RG, Neil J, Dierker D, Smyser CD, Wallendorf M, Kidokoro H, et al. Alterations in Brain Structure and Neurodevelopmental Outcome in Preterm Infants Hospitalized in Different Neonatal Intensive Care Unit Environments. The Journal of pediatrics. 2014;164(1):52-60. e2.

5.Flacking R, Lehtonen L, Thomson G, Axelin A, Ahlqvist S, Moran VH, et al. Closeness and separation in neonatal intensive care. Acta Paediatrica. 2012.

6.Lehtonen L, Axelin, A., Raiskila,S., Ahlqvist-Björkroth, S. and the SCENE group. The international Closeness Survey in the SCENE Study 2013.

7.Gooding JS, Cooper LG, Blaine AI, Franck LS, Howse JL, Berns SD, editors. Family support and family-centered care in the neonatal intensive care unit: origins, advances, impact2011: Elsevier.

8.Marzieh S. Impact of the Design of Neonatal Intensive Care Units on Neonates, Staff, and Families. 2012.

9.Ortenstrand A, Westrup B, Brostrom EB, Sarman I, Akerstrom S, Brune T, et al. The Stockholm Neonatal Family Centered Care Study: effects on length of stay and infant morbidity. Pediatrics. 2010:peds. 2009-1511v1.

10.Pineda RG, Stransky KE, Rogers C, Duncan MH, Smith GC, Neil J, et al. The single-patient room in the NICU: maternal and family effects. Journal of Perinatology. 2011.

11.Cattaneo A, Davanzo R, Uxa F, Tamburlini G. Recommendations for the implementation of kangaroo mother care for low birthweight infants. Acta Paediatrica. 1998;87(4):440-5.

12. Conde-Agudelo A, Diaz-Rossello JL, Belizan JM. Kangaroo mother care to reduce morbidity and mortality in low birthweight infants. Cochrane Database Syst Rev. 2003;2(2).

13.Cong X, Cusson RM, Walsh S, Hussain N, Ludington-Hoe SM, Zhang D. Effects of Skin-to-Skin Contact on Autonomic Pain Responses in Preterm Infants. The Journal of Pain. 2012.

14.Blomqvist YT, Rubertsson C, Kylberg E, Jöreskog K, Nyqvist KH. Kangaroo Mother Care helps fathers of preterm infants gain confidence in the paternal role. Journal of Advanced Nursing. 2011.

15.Dodd VL. Implications of kangaroo care for growth and development in preterm infants. Journal of Obstetric, Gynecologic, & Neonatal Nursing. 2005;34(2):218-32.

16.Klingenberg C, Embleton ND, Jacobs SE, O'Connell LAF, Kuschel CA. Enteral feeding practices in very preterm infants: an international survey. Archives of Disease in Childhood-Fetal and Neonatal Edition. 2012;97(1):F56-F61.

17.World Health O. Indicators for Assessing Infant and Young Child Feeding Practices: Conclusions of a Consensus Meeting Held 6-8 November 2007 in Washington DC, USA: World Health Organization (WHO); 2008.

18.Meijssen DE, Wolf MJ, Koldewijn K, Van Wassenaer AG, Kok JH, Van Baar AL. Parenting stress in mothers after very preterm birth and the effect of the Infant Behavioural Assessment and Intervention Program. Child: Care, Health and Development. 2011.

19.Docherty SL, Miles MS, Holditch-Davis D. Worry about child health in mothers of hospitalized medically fragile infants. Advances in neonatal care. 2002;2(2):84.

20.Holditch Davis D, Bartlett TR, Blickman AL, Miles MS. Posttraumatic stress symptoms in mothers of premature infants. Journal of Obstetric, Gynecologic, & Neonatal Nursing. 2003;32(2):161-71.

21.Howland LC, Pickler RH, McCain NL, Glaser D, Lewis M. Exploring Biobehavioral Outcomes in Mothers of Preterm Infants. MCN: The American Journal of Maternal/Child Nursing. 2011;36(2):91.

22.Miles MS, Holditch-Davis D, Schwartz TA, Scher M. Depressive symptoms in mothers of prematurely born infants. Journal of Developmental & Behavioral Pediatrics. 2007;28(1):36.

23.Franck LS, Cox S, Allen A, Winter I. Measuring neonatal intensive care unit‐related parental stress. Journal of Advanced Nursing. 2005;49(6):608-15.

24.Joseph RA, Mackley AB, Davis CG, Spear ML, Locke RG. Stress in fathers of surgical neonatal intensive care unit babies. Advances in neonatal care. 2007;7(6):321.

25.Lundqvist P, Jakobsson L. Swedish men's experiences of becoming fathers to their preterm infants. Neonatal network: NN. 2003;22(6):25.

26.Ravn IH, Lindemann R, Smeby NA, Bunch EH, Sandvik L, Smith L. Stress in fathers of moderately and late preterm infants: a randomised controlled trial. Early Child Development and Care. 2011;99999(1):1-16.

27.Tandberg BS, Sandtrø, H.P, Vårdal, M., Rønnestad, A. . Parents of preterm evaluation of stress and nursing support. Journal of Neonatal Nursing. 2013;IN PRESS. <http://dx.doi.org/10.1016/j.jnn.2013.01.008>.

28.Feeley N, Waitzer E, Sherrard K, Boisvert L, Zelkowitz P. Fathers’ perceptions of the barriers and facilitators to their involvement with their newborn hospitalised in the neonatal intensive care unit. Journal of clinical nursing. 2013;22(3-4):521-30.

29.Meyer EC, Coll CTG, Lester BM, Boukydis CFZ, McDonough SM, Oh W. Family-based intervention improves maternal psychological well-being and feeding interaction of preterm infants. Pediatrics. 1994;93(2):241-6.

30.Melnyk BM, Crean HF, Feinstein NF, Fairbanks E. Maternal anxiety and depression following a premature infants’ discharge from the NICU: explanatory effects of the COPE program. Nursing Research. 2008;57(6):383.

31.Dennis CL. The Breastfeeding Self‐Efficacy Scale: Psychometric Assessment of the Short Form. Journal of Obstetric, Gynecologic, & Neonatal Nursing. 2003;32(6):734-44.

32.Haga SM. Identifying risk factors for postpartum depressive symptoms: the importance of social support, self-efficacy, and emotion regulation. 2012.

33.Wheeler BJ, Dennis CL. Psychometric Testing of the Modified Breastfeeding Self‐Efficacy Scale (Short Form) Among Mothers of Ill or Preterm Infants. Journal of Obstetric, Gynecologic, & Neonatal Nursing. 2013;42(1):70-80.

34.Miles MS, Funk SG, Carlson J. Parental Stressor Scale: neonatal intensive care unit. Nursing Research. 1993;42(3):148-52.

35.Cox JL, Holden JM, Sagovsky R. Detection of postnatal depression. Development of the 10-item Edinburgh Postnatal Depression Scale. The British journal of psychiatry. 1987;150(6):782-6.

36.Eberhard-Gran M, Eskild A, Tambs K, Schei B, Opjordsmoen S. The Edinburgh postnatal depression scale: validation in a Norwegian community sample. Nordic Journal of Psychiatry. 2001;55(2):113-7.

37.Eberhard‐Gran M, Eskild A, Tambs K, Opjordsmoen S, Ove Samuelsen S. Review of validation studies of the Edinburgh Postnatal Depression Scale. Acta Psychiatrica Scandinavica. 2001;104(4):243-9.

38.Spielberger CD. State‐trait anxiety inventory: Wiley Online Library; 2005.

39.Marteau TM, Bekker H. The development of a six‐item short‐form of the state scale of the Spielberger State—Trait Anxiety Inventory (STAI). British Journal of Clinical Psychology. 1992;31(3):301-6.

40.Condon JT, Dunn DJ. Nature and determinants of parent-to-infant attachment in the early postnatal period. Journal of the American Academy of Child & Adolescent Psychiatry. 1988;27(3):293-9.

41.Abidin RR. Parenting Stress Index: Manual, Administration Booklet,[and] Research Update. 1983.

42.Abidin RR, Flens JR, Austin WG. Parenting Stress Index. Forensic uses of clinical assessment instruments. 1995:297-328.

43.Kaaresen PI, Rønning JA, Ulvund SE, Dahl LB. A randomized, controlled trial of the effectiveness of an early-intervention program in reducing parenting stress after preterm birth. Pediatrics. 2006;118(1):e9-e19.

44.Kornør H, & Martinussen, M. . . PSI: Parenting Stress Index, 3. versjon. I H. Kornør (red.), PsykTestBARN. Hentet fra <http://www.psyktest.no/PSI.63155.cms>. . 2011.
